# Unveiling Common Bean (*Phaseolus vulgaris* L) RNA‐ and DNA‐Based Virome in Western Kenya: Insights From Metatranscriptomic and Metagenomic Signatures

**DOI:** 10.1155/av/6690945

**Published:** 2025-10-29

**Authors:** Aggrey Keya Osogo, Francis Muyekho, Hassan Were, Patrick Okoth

**Affiliations:** ^1^ Department of Biological Sciences, Masinde Muliro University of Science and Technology, P.O. Box 190, Kakamega, 50100, Kenya, mmust.ac.ke; ^2^ Department of Biological and Environmental Science, Kibabii University, P.O. Box 1699, Bungoma, 50200, Kenya, kibabii-university-public-university.business.site; ^3^ Department of Agriculture and Land Use Management, Masinde Muliro University of Science and Technology, Kakamega, Kenya, mmust.ac.ke

**Keywords:** common beans, DNA-based virome, metagenomic signatures, metatranscriptomic, RNA

## Abstract

Common bean (*Phaseolus vulgaris* L) is Kenya’s second most important agricultural product after maize, serving as a vital source of protein for many rural families in Western Kenya. However, viral diseases caused by RNA and DNA viruses greatly impair bean productivity, often leading to yield losses of up to 100%, thus contributing to food insecurity. Global research has isolated 168 viruses of plants that have detrimental effects on common beans; however, no extensive profiling of these viruses has been done in Western Kenya. The scope of this study was to delineate the whole virome that infects common beans through a comprehensive disease diagnostic survey. Sixty‐one diseased samples were collected, and nucleic acids were extracted using standard extraction protocols (DNA &RNA Qiagen) and sequenced on the Illumina platform. Metagenomic analysis revealed several DNA‐based viruses, such as *Badnavirus* spp, *Caulimovirus maculatractylodei*, *Pandanus badnavirus*, *Okra enation leaf curl virus*, and *Paper mulberry vein-banding virus*, while metatranscriptomic analysis uncovered viruses like *Tomato leaf curl Cameroon alphasatellite*, *Physalis Rugose Mosaic Virus*, Citrus endogenous paretrovirus, *Natevirus nate*, and *Bracoviriform facetosae*. To the best of our knowledge, this study provides a comprehensive inventory of viral entities associated with common beans not documented in Africa. This information is essential for defining plant defense mechanisms, guiding crop protection strategies, lowering agriculture‐related risks, strengthening resistance, and advancing resilience.

## 1. Introduction

Common bean (*Phaseolus vulgaris*) is one of the most widely cultivated legumes worldwide, providing a vital source of protein, predominantly in sub‐Saharan Africa. In Western Kenya, beans are a major protein source and a key diet component for many rural households. However, the productivity of common beans is significantly hindered by various biotic stressors, especially viral diseases. These diseases are often caused by RNA and DNA viruses that infect common bean plants, causing yield losses of up to 100% [1, 2], diminished seed quality, and in some cases, whole crop failure. The global landscape of viruses infecting common beans is complex, with significant implications for agricultural productivity. Research indicates that a total of 168 plant viruses from 39 genera and 16 families have been documented to infect common beans, highlighting the extensive viral diversity affecting this crop [3].

Viral diseases of common beans are a significant concern for legume production worldwide, with several viruses identified as major threats to crop health. Among the most notable are the bean common mosaic virus (BCMV), bean golden mosaic virus (BGMV), and bean yellow mosaic virus (BYMV). A thorough understanding of the complete virome affecting or associated with common beans, as well as their characteristics and management strategies, is essential for effective control. BCMV is caused by two closely related potyviruses: BCMV and bean common mosaic necrosis virus (BCMNV), which affect common beans globally [4]. In addition, isolates from South and Southeast Asia exhibit high genetic diversity, with sequence identities ranging from 92.1% to 98.8% [5]. Transmission of these viruses via infected seeds can be as high as 38.1% [5]. Worldwide, other viruses have been documented to affect legumes, including the BYMV and the cucumber mosaic virus (CMV), both of which have a global distribution and pose significant challenges to legume crops [6]. Studying the total virome in common beans is therefore fundamental to understanding viral diversity, improving crop resilience, and ensuring food security. The emergence of new viral strains poses significant threats to bean production globally, necessitating comprehensive surveillance and diagnostic techniques. High‐throughput sequencing (HTS) has revealed a variety of viruses affecting common beans, including BCMV and BCMNV, with new strains being identified in regions such as Kenya and India [7, 8].

Studies documenting common bean viruses in Kenya are limited, primarily relying on serological techniques [9, 10]. However, this method has major pitfalls as each antibody detects only a specific virus or a closely related group, potentially leading to false negatives in symptomatic samples. Recent advancements in molecular biology, particularly metatranscriptomics and metagenomics, offer more comprehensive tools for identifying and characterizing the diversity of pathogens in crops [11]. These HTS techniques enable the identification of known and novel viruses affecting the crop, providing a broader view of the viral landscape and their interactions with the plant host [12, 13]. In Kenya, the first application of metagenomic studies on common bean germplasm in Central Kenya detected BCMNV, CMV, and *P. vulgaris* alpha endornavirus (PvEV) in common beans [7, 14]. This study aimed to leverage metatranscriptomic and metagenomic approaches to reveal the full range of RNA‐based and DNA viruses that infect common beans in Western Kenya. This information is necessary for developing more effective and sustainable strategies to combat viral infections in beans, which will eventually contribute to improved agricultural output and enhanced food security for susceptible populations in Western Kenya and beyond.

## 2. Materials and Methods

Surveys aimed at diagnosing common bean viruses were conducted in April 2024 within the Western region of Kenya, which shares a border with Uganda. The area is administratively subdivided into Kakamega (0°17′3.19″ N 34°45′8.24″ E), Busia, (0.3416°N, 34.3352°E), Vihiga (0° 3′ 0″ N 34° 49′ 0″ E 0.05, 34.81667), Bungoma, (0.6595°N, 0 34.5639°E), and Nandi (0.0958°N, 34.8679°E) counties adopted from Reference [15] (Figure 1(a)) using coordinates (SM3). Temperatures characterize these areas range from a minimum of 14°C–18°C to a maximum of 30°C–36°C throughout the year. The heaviest rainfall is in April and the lowest in January. Average annual precipitation ranges from 1740 mm to 1940 mm. A total of 61 leaf samples of common bean, showing symptoms like mosaic, vein banding, leaf curling, chlorosis, and necrosis (Figures 1(b), 1(c), 1(d), 1(e), 1(f), 1(g)), were collected from Bungoma (13), Kakamega (12), Nandi (12), Busia (12), and Vihiga (12). The samples were placed in falcon tubes and stored in a container with liquid nitrogen.

Figure 1(a) Map of the Western Kenya region highlighting the specific areas where samples were collected in various counties and sub‐counties adopted from Reference [15]. (b) Diseased common bean plant showing necrosis, leaf curling, and mottling collected from Kabuchai, Bungoma County. (c) Diseased common bean showing leaf mosaic and necrosis collected from Sirende, Bungoma County. (d) Diseased common bean showing extensive leaf mosaic and yellowing collected from Mukulusu Kakamega County. (e) Diseased common bean showing extensive leaf curling and necrotic spots from Butula, Busia County. (f) Diseased common bean showing extensive yellowing collected from Bukura, Kakamega County. (g) Diseased common bean showing yellowing, necrotic spots, and leaf curling collected from Musasa, Vihiga County.

**Figure:**
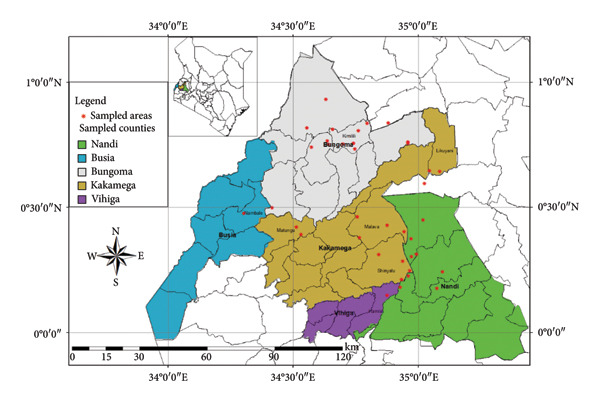
(a)

**Figure:**
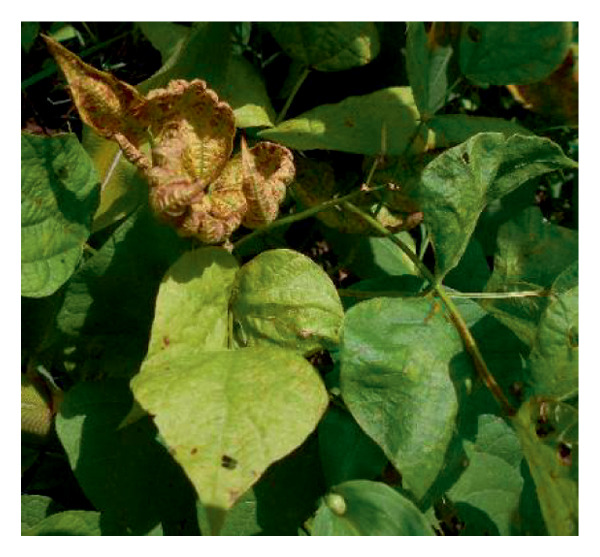
(b)

**Figure:**
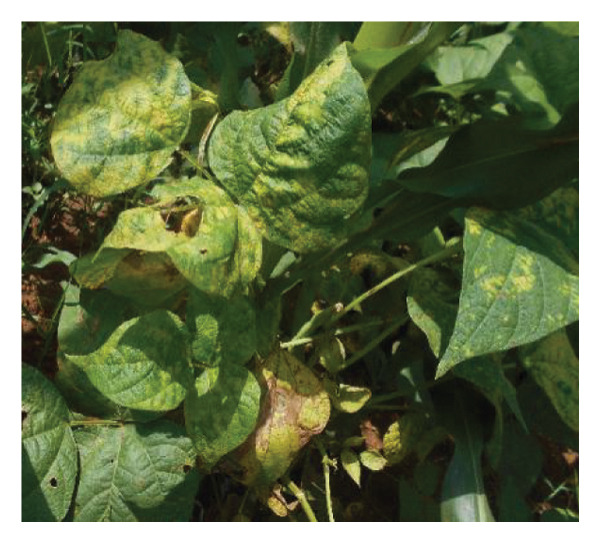
(c)

**Figure:**
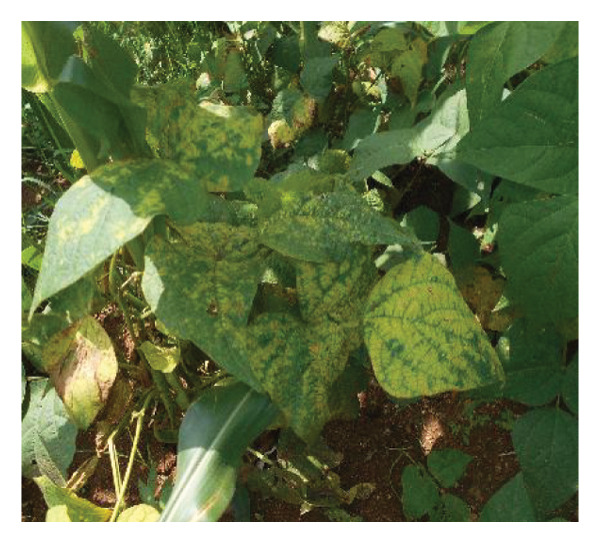
(d)

**Figure:**
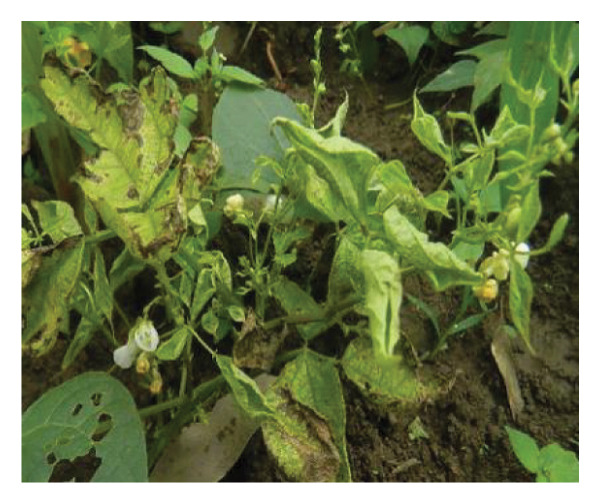
(e)

**Figure:**
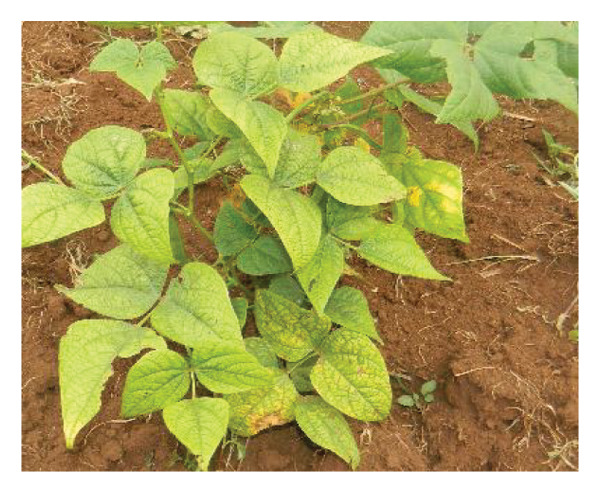
(f)

**Figure:**
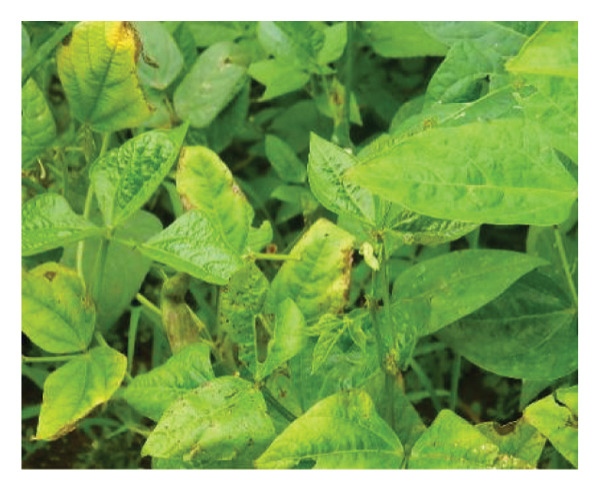
(g)

The samples were then preserved in a −80°C freezer to maintain their integrity until the appropriate time for total DNA and RNA extraction. Following the extraction of nucleic acids, three composite DNA samples were successfully obtained.

### 2.1. Total DNA and RNA Extraction

For DNA extraction, approximately 100 mg of juvenile foliar tissue was procured and subsequently positioned in a 2‐mL safe‐lock microcentrifuge tube with a tungsten carbide bead. The tissue underwent disruption and was subsequently transferred to a precooled adapter within a Mixer Mill homogenizer, where it was pulverized for 1 min at a frequency of 25 Hz. Upon grinding the tissue into a fine powder, the established protocol for the Qiagen DNeasy Plant Mini Kit exactly adhered to extract the genomic DNA; as per the manufacturer’s guidelines, DNA was isolated from samples collected in Kakamega, Nandi, Bungoma, Vihiga, and Nandi counties, which were mixed to generate three samples (DVKI, DBGM1, and DBU1).

For RNA extraction, the 61 common bean leaf samples collected were ground in liquid nitrogen. Total RNA was extracted using RNeasy Mini Kits (Qiagen) with proteinase K (Qiagen), lysozyme (Sigma‐Aldrich), and DNAse I (Sigma‐Aldrich). RNA concentration was measured using a NanoDrop 2000 spectrophotometer (Thermo Scientific), and RNA integrity was assessed using a Tape Station 2200 electrophoresis system (Agilent Technologies) to determine the RNA integrity number (RIN). Samples were selected for RNA‐seq based on an rRNA ratio > 0.8 and RIN > 8. The samples were pooled to form three composite samples based on the location RBGM1 (Bungoma), RBU1 (Busia), and RVK1 (Vihiga/Kakamega).

**Figure 2:**
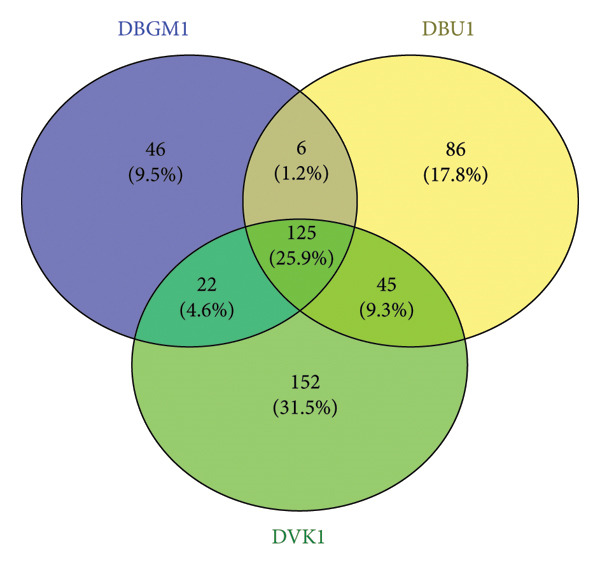
Venn diagram showing the sample identities, count, and percentages of DNA viruses unique to and commonly shared among Bungoma, Busia, and Vihiga/Kakamega counties.

For DNA analysis, common bean leaf samples exhibiting disease‐like symptoms, such as severe or mild mosaic, vein banding, leaf curling, yellowing, and necrosis (Figure 2), were collected in falcon tubes and promptly placed in a container with liquid nitrogen. The samples were stored in an 80°C freezer to preserve their integrity until they were ready for total DNA extraction. Among the 91 samples collected, only 16 exhibited mixed infections of BCMV and BCMNV infections (Kakamega‐3, Nandi 3, Bungoma 4, Vihiga 4, and Nandi 2) and were selected for DNA extraction using the DNeasy Kit according to the manufacturer’s instructions. The samples were pooled to form three composite samples based on the location DBGM1 (Bungoma), DBU1 (Busia), and DVK1 (Vihiga/Kakamega).

### 2.2. Library Construction of DNA and RNA, Quality Control (QC), and Sequencing

All the pooled samples for DNA and RNA analysis were sent to Novogene Limited Company in Singapore for deep sequencing, with QC checks performed before library construction and sequencing.

DNA libraries were prepared by taking 1 μg of genomic DNA from the samples mentioned earlier and randomly breaking it into pieces about 350 bp long using a Covaris ultrasonic disruptor. The library creation involved several important steps: end repair, A‐tailing, ligating sequencing adapters, purification, and PCR amplification. After constructing the library, its integrity and the size of the insert fragments were checked via AATI analysis. After a thorough QC, different libraries were pooled based on their concentrations and expected data needs, then they were sequenced using PE150 technology.

RNA library preparation involved the selective removal of ribosomal RNAs from both eukaryotic and prokaryotic organisms in the total RNA samples. The residual RNAs were subjected to fragmentation, yielding fragments of approximately 250–300 base pairs, and subsequently reverse‐transcribed into double‐stranded complementary DNAs, which underwent end repair, A tailing, and adapter ligation. After selecting fragment sizes and PCR amplification, the metatranscriptome library was prepared for QC and sequencing procedures. The library underwent quantification utilizing Qubit and real‐time PCR methodologies, along with an assessment of size distribution via bioanalyzer techniques. The quantified libraries were combined and sequenced under the effective library concentrations and the requisite volume of data.

### 2.3. Analysis of the Metatranscriptome

Trinity, developed by the Broad Institute and the Hebrew University of Jerusalem, is a transcriptome assembler comprising three modules: Inchworm, Chrysalis, and Butterfly. Inchworm generates contigs from k‐mers (*k* = 25), Chrysalis clusters these contigs into de Bruijn graphs representing splice variants or paralogs, and Butterfly reconstructs full‐length transcripts by integrating the graphs with the original reads, producing the final *TRINITY.fasta* output. For transcript clustering, Corset (https://github.com/Oshlack/Corset/wiki) sorts transcripts by length and groups them based on sequence similarity, generating clusters when similarity falls below a defined cutoff. Unigene sequences were aligned to the Micro_NR database, which includes bacteria, fungi, archaea, and viruses from NCBI’s NR, using DIAMOND (https://github.com/bbuchfink/diamond/) [16] with the blastp algorithm and an E‐value cutoff of 1e − 5 [17]. The Lowest Common Ancestor (LCA) algorithm in MEGAN [18] was then applied for taxonomic annotation. Species and higher‐level taxonomic abundances were derived from annotated gene counts [19–21], and MetaStat was used to perform permutation‐based group comparisons at each taxonomic level.

To investigate the evolutionary relationships of viral sequences, orthologous sequences encoding reverse transcriptase were selected for phylogenetic analysis. These sequences were aligned using MAFFT v7.525 with default parameters. A Bayesian phylogenetic tree was constructed using BEAST2 v2.5.0. The best‐fitting substitution model was selected based on Bayesian Information Criterion (BIC) using ModelFinder, and a Markov Chain Monte Carlo (MCMC) analysis was run for 10 million generations to ensure convergence. Trees were sampled every 1000 generations, and the first 10% of the sampled trees were discarded as burn‐in. The final maximum clade credibility (MCC) tree was generated using TreeAnnotator and visualized using Figtree v1.4.4, where branch annotations and labeling were applied to enhance interpretability. Tree Figure legend: Posterior probabilities are depicted as node colors, with a corresponding legend indicating their values to represent branch/clade support. Higher posterior probability values indicate stronger statistical confidence in the branching structure, while lower values suggest weaker support.

### 2.4. Metagenome Assembly

Raw metagenomic (DNA) sequencing reads were first processed to remove host‐derived sequences by mapping them to the *P. vulgaris* reference genome using HISAT2 v2.2.1. Unmapped reads were retained for downstream viral analysis. The filtered reads were then classified taxonomically using Kraken2 v2.1.3 against the RefSeq Viral database (https://benlangmead.github.io/aws-indexes/k2) to identify viral sequences. To refi netaxonomic assignments and estimate relative viral abundance, Bracken v3.0 was used to generate an abundance matrix from the Kraken2 output. The viral abundance profiles were visualized as heatmaps using pandas, matplotlib, and seaborn packages in Python v3.8.12. Core viral species shared across different samples were identified and visualized using Venny v2.1.0 (https://bioinfogp.cnb.csic.es/tools/venny/).

Metagenomic reads were assembled using ViralSPAdes in SPAdes v4.0.0. The assembled contigs were mapped back to the RefSeq Viral database to ensure only viral sequences were retained, and nonviral contigs were discarded. Gene prediction was performed on the filtered viral contigs using Prodigal v2.6.3 to identify open reading frames (ORFs). The predicted proteins were then categorized into orthologous groups using OrthoFinder v2.2.7. However, due to the limited data in the metagenomic dataset, OrthoFinder failed to group DNA‐based viral proteins into orthologous clusters.

To investigate the evolutionary relationships of viral sequences, orthologous sequences encoding reverse transcriptase were selected for phylogenetic analysis. These sequences were aligned using MAFFT v7.525 with default parameters. A Bayesian phylogenetic tree was constructed using BEAST2 v2.5.0. The best‐fitting substitution model was selected based on BIC using ModelFinder, and an MCMC analysis was run for 10 million generations to ensure convergence. Trees were sampled every 1000 generations, and the first 10% of the sampled trees were discarded as burn‐in. The final MCC tree was generated using TreeAnnotator and visualized using Figtree v1.4.4, where branch annotations and labeling were applied to enhance interpretability. Tree Figure legend: Posterior probabilities are depicted as node colors, with a corresponding legend indicating their values to represent branch/clade support. Higher posterior probability values indicate stronger statistical confidence in the branching structure, while lower values suggest weaker support.

## 3. Results

### 3.1. Metagenome

The metagenomic assembly of the three samples produced 470,069,181 high‐quality reads for Vihiga DVK1, 195,073,555 high‐quality reads for Busia (DBU1), and 364,374,455 high‐quality reads for Bungoma (DBGM1), which have been deposited in the NCBI database (https://www.ncbi.nlm.nih.gov/sra/PRJNA1226238). The quality of the reads were as in SM1.

Upon removal of host reads and other eukaryotic organisms, the viral contigs were distributed as follows: 25.9% of the viral organisms were commonly expressed across all regions studied, while 9.5%, 17.8%, and 31.5% were unique to Bungoma, Busia, and Vihiga/Kakamega, respectively (Figure 2).

Stilbocarpa mosaic bacilliform virus, Caulimovirus maculatractylodei, and Okra enation leaf curl virus occurred in all the counties. The Vihiga (RVK1) sample contained solely unique viruses, including *P. vulgaris* alpha endornavirus 3, and Badnavirus *betananas*, as viruses affecting common bean (SM 2).

**Figure 3:**
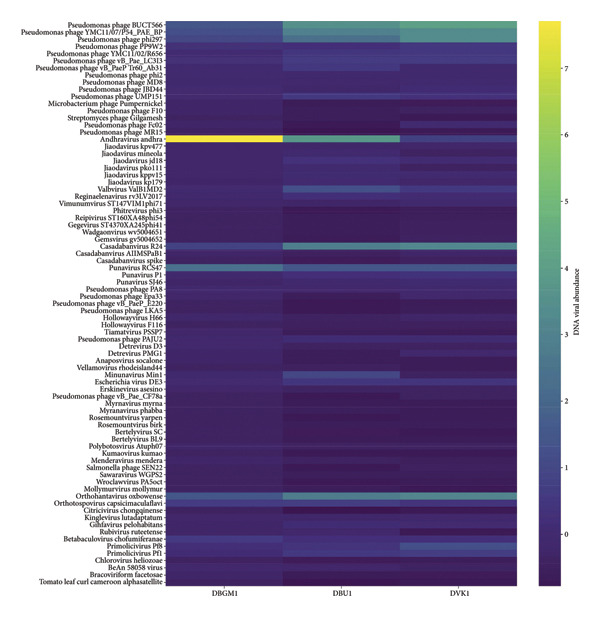
Heat map displaying the Viral Taxa on the *Y*‐axis (left side) and DNA viral abundance on the *X*‐axis, with sample identities for Bungoma (DBGM1), Busia (DBU1), and Kakamega/Vihiga (DVK1), along with their corresponding relative abundances. The viral abundance values were normalized using StandardScaler from scikit‐learn, which standardizes data by subtracting the mean and scaling to unit variance for each feature. This transformation ensured that all features contributed equally to the abundance and heat map visualization, preventing samples with higher sequencing depth or extreme values from dominating the analysis. The units used for classification were viral contigs.

After analysis, the contigs were matched to 93 viral species, 30 genera, 47 unclassified genera, 16 viral families, and 48 unclassified families SM 2. The majority of the DNA viruses were from the genus *Badnavirus*, which included *Badnavirus betacolocalasiae*, *Badnavirus phirubi*, *Badnavirus venatheobromae*, *Jujube-associated badnavirus*, *Pandanus badnavirus*, *Paper mulberry vein-banding virus*, *Pelargonium vein-banding virus*, and *Stilbocarpa mosaic bacilliform virus*. Other identified viruses included *Caulimovirus maculatractylodei*, *Atrato Retro-like virus*, *Okra enation leaf curl virus*, *Cotesia sesamiae bracovirus*, *Pandoravirus massiliensis*, *Lanavirus lana*, *Bucovirus buco*, among others (Table 1 and Figure 3). The Bungoma samples exhibited the highest relative abundance of *Punavirus*, while the Busia and Vihiga/Kakamega samples showed the highest relative abundance of *Cadabanvirus R24* and *Orthohantavirus oxbowaense* viruses.

**Table 1:** Viral sequences representing viruses infecting common beans, including their viral genera and families.

	Virus species	Genera	Unigenes			Family
			DVK1	DBU1	DBGM1	
1	Badnavirus betacolocalasiae	Badnavirus; s	58.925778	0	0	Caulimoviridae
2	Badnavirus phirubi	Badnavirus; s	33.104216	0	0	Caulimoviridae
3	Badnavirus venatheobromae	Badnavirus; s	18.046191	0	0	Caulimoviridae
4	Jujube associated badnavirus	Badnavirus; s	51.560055	0	0	Caulimoviridae
5	Pandanus badnavirus	Badnavirus; s	85.581874	0	0	Caulimoviridae
6	Paper mulberry vein‐banding virus	Badnavirus; s	171.40291	0	0	Caulimoviridae
7	Pelargonium vein banding virus	Badnavirus; s	30.365742	0	0	Caulimoviridae
8	Stilbocarpa mosaic bacilliform virus	Badnavirus; s	36.912123	70.04472	30.18612	Caulimoviridae
9	Caulimovirus maculatractylodei	Caulimovirus; s	52.58102	0	0	Caulimoviridae
10	Okra enation leaf curl virus	Begomovirus; s	124.23145	137.4403	251.8785	Geminiviridae

### 3.2. Metatranscriptome

The raw sequences derived from the transcriptome were deposited in a public repository (https://www.ncbi.nlm.nih.gov/sra/PRJNA1226238) (NCBI database) for reference. The raw reads for RVKI, RBU1, and RBGM1 were 37,047,327, 35,629,275, and 44,496,699, respectively. After cleaning, the reads were reduced to 35,716,390, 34,974,467, and 44,004,580 for RVKI, RBU1, and RBGM1, respectively. The RVKI, RBU1, and RBGM1 error rates were 0.03, 0.02, and 0.02, respectively. The high‐quality reads obtained from all three samples enabled the subsequent taxonomic and functional annotation.

**Figure 4:**
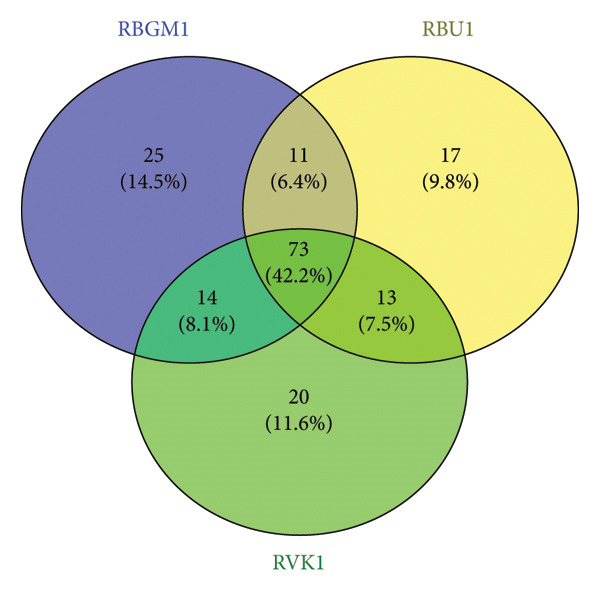
Venn diagram showing the sample identities, count, and percentages of RNA viruses unique to and commonly shared among Bungoma, Busia, and Vihiga/Kakamega counties.

Following the removal of host reads and other eukaryotic organisms, the viral contigs were distributed as follows: 42.2% of the viral organisms were commonly expressed across the regions studied, while 25%, 17%, and 20% were unique to Bungoma, Busia, and Vihiga/Kakamega, respectively (Figure 4).

**Figure 5:**
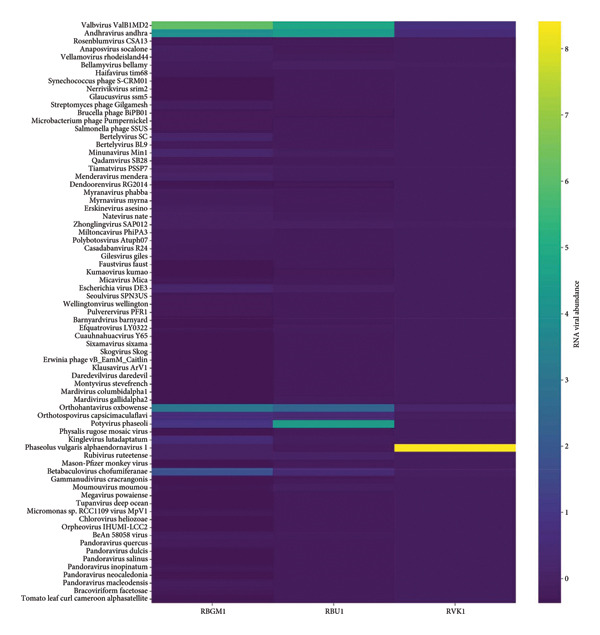
Heat map displaying the Viral Taxa on the *Y*‐axis (left side) and RNA viral abundance on the *X*‐axis, with sample identities for Bungoma (DBGM1), Busia (DBU1), and Kakamega/Vihiga (DVK1), along with their corresponding relative abundances. The viral abundance values were normalized using StandardScaler from scikit‐learn, which standardizes data by subtracting the mean and scaling to unit variance for each feature. This transformation ensured that all features contributed equally to the relative abundance and heat map visualization, preventing samples with higher sequencing depth or extreme values from dominating the analysis.

**Figure 6:**
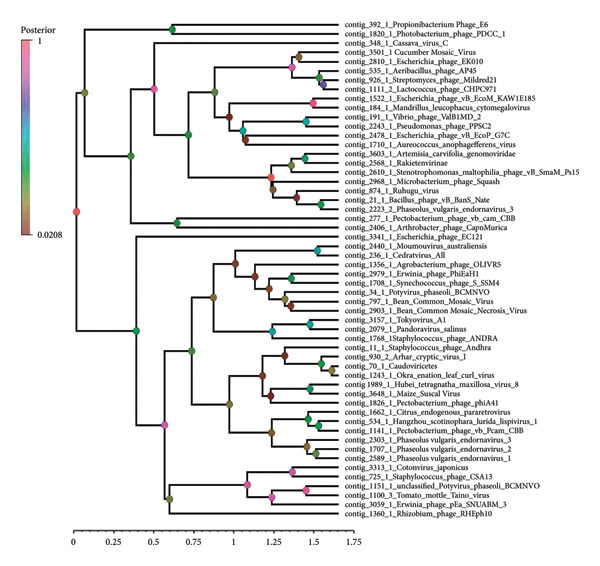
A phylogenetic tree constructed using BEAST2 v2.5.0. The best‐fitting substitution model was chosen based on the Bayesian Information Criterion (BIC) via ModelFinder, and a Markov Chain Monte Carlo (MCMC) analysis was conducted for 10 million generations to ensure convergence. Trees were sampled every 1000 generations, with the first 10% of the trees discarded as burn‐in. The final maximum clade credibility (MCC) tree was produced using TreeAnnotator and visualized with Figtree v1.4.4, where branch annotations and labels were added to improve interpretability. Tree figure legend: Posterior probabilities are represented by node colors, with a corresponding legend indicating their values to illustrate branch/clade support. Higher posterior probability values indicate stronger confidence in the branching structure, while lower values suggest weaker support.

Analysis of the transcriptome showed that the contigs mapped to 74 viruses in the NCBI database, including *Potyvirus phaseoli*, Tomato leaf curl Cameroon alphasatellite, *P. vulgaris endornavirus*, *Physalis Rugose Mosaic Virus*, CMV, Maize Suscal Virus, Okra enation leaf curl virus, Barnyard virus barnyard, Citrus endogenous paretrovirus, Tiamat virus, *Natevirus nate*, *Myrnavirus myrna*, *Betabaculovirus chofumeferanae*, *Minunavirus*, *Orthohantavirus oxbowaense*, *Bracoviriform facetosae*, Holloway virus, Berteley virus, *Kuma virus*, and others (Figure 5). The relative abundance of *Valbvirus ValBIMD2* was highest in Bungoma, followed by Busia samples. *Andhravirus andhra* was most abundant in both Bungoma and Busia samples. *Potyvirus phaseoli* (BCMNV0) showed the highest relative abundance in Busia samples, while *P. vulgaris endornavirus 1* was most prevalent in the Vihiga/Kakamega samples. A phylogenetic tree was constructed of the viral taxa, indicating their evolutionary relationship (Figure 6).

**Figure 7:**
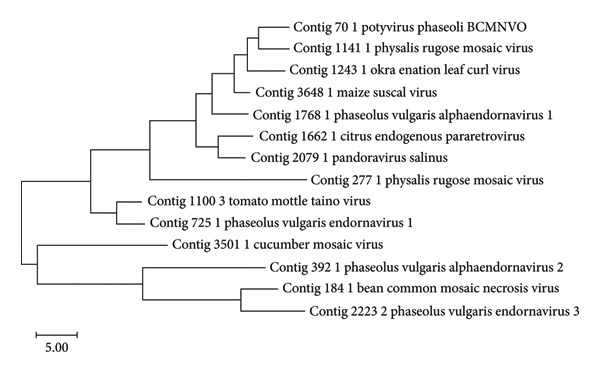
Evolutionary analysis by the maximum likelihood method.

The Bayesian phylogenetic tree reconstructed the evolutionary relationships among radically diverse viruses and bacteriophages, branch lengths indicating genetic divergence, and node colors indicating posterior probability support (Figure 6). The close relatives of viruses were clustered together as a group, for instance, plant viruses such as the CMV constituted discrete clades. The purple‐to‐green to‐red color gradient represents confidence in the branching, where higher posterior values (purple/blue) indicated high support. Longer branches represent more evolutionary change, and shorter branches indicate recent divergences. The tree reveals deep insight into viral taxonomy with shared ancestry, evolutionary distances, and potential adaptations specific to hosts. Furthermore, the viruses were filtered to include only the important ones, both known and unknown, that infect common beans (Figure 7). In addition, we further compared the viruses Physalis rugose mosaic virus with NCBI accession numbers Figure 8(a) and *P. vulgaris* alpha endornavirus with NCBI accession numbers and constructed a phylogenetic tree (Figure 8(b)).

Figure 8(a) Evolutionary relationship of 3 taxa: The evolutionary history was inferred using the neighbor‐joining method [23]. The optimal tree with the sum of branch length = 4.242 is shown. The evolutionary distances were computed using the Poisson correction method [26] and are expressed in units of amino acid substitutions per site. The analytical procedure encompassed 3 amino acid sequences. The pairwise deletion option was applied to all ambiguous positions for each sequence pair, resulting in a final data set comprising 522 positions. Evolutionary analyses were conducted in MEGA12 [25]. (b) Evolutionary analysis by the maximum likelihood method: The phylogeny was inferred using the maximum likelihood method and Jones–Taylor–Thornton (1992) model [22] of amino acid substitutions, and the tree with the highest log likelihood (−14,425.36) is shown. The initial tree for the heuristic search was selected by choosing the tree with the superior log‐likelihood between a neighbor‐joining (NJ) tree [23] and a maximum parsimony (MP) tree. The NJ tree was generated using a matrix of pairwise distances computed using the p‐distance [23]. The MP tree had the shortest length among 10 MP tree searches, each performed with a randomly generated starting tree. The analytical procedure encompassed 4 amino acid sequences with 4852 positions in the final dataset. Evolutionary analyses were conducted in MEGA12 [25] (4) utilizing up to 6 parallel computing threads.

**Figure:**
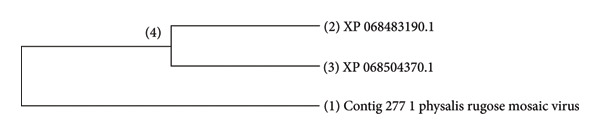
(a)

**Figure:**
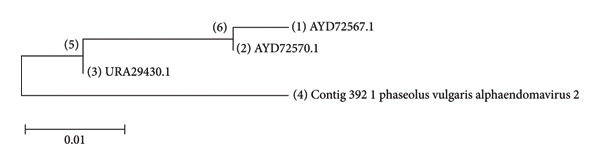
(b)

The phylogeny was inferred using the maximum likelihood method and the Jones–Taylor–Thornton (1992) model [22] of amino acid substitutions. The tree with the highest log likelihood (−1824.47) is shown. The initial tree for the heuristic search was selected by choosing the tree with the superior log‐likelihood between a neighbor‐joining (NJ) tree [23] and a maximum parsimony (MP) tree. The NJ tree was generated using a matrix of pairwise distances computed using the p‐distance [24]. The MP tree had the shortest length among 10 MP tree searches, each performed with a randomly generated starting tree. The analytical procedure encompassed 14 amino acid sequences with 211 positions in the final dataset. Evolutionary analyses were conducted using MEGA12 [25], which utilized up to six parallel computing threads.

## 4. Discussion

This study provides the first integrated characterization of RNA‐ and DNA‐based viruses infecting common bean (*P. vulgaris* L.) in Western Kenya using metatranscriptomic and metagenomic approaches. The spatial distribution of viral contigs across the studied regions reveals informative trends in the virome of common bean agro‐ecosystems in Western Kenya. Notably, a substantial percentage of viral entities exhibited universal expression across all the regions investigated, indicating that the viruses are highly prevalent and show a high degree of adaptation to varied agro‐ecological situations. The widespread distribution of these viruses may be attributed to general agronomic practices, seed swaps, or the shifting of insect vectors that facilitate virus transmission across different regions [27].

Using metatranscriptomic technology, this study uncovered a wide array of viruses, with contigs mapping to 74 different viruses in the NCBI database. These viruses belonged to various families and genera, encompassing diverse plant and animal pathogens. Among the identified viruses were *Potyvirus phaseoli* (=BCMNV), Tomato leaf curl Cameroon alphasatellite, *P. vulgaris* endornavirus, Physalis Rugose Mosaic Virus, CMV, Maize suscal virus, Okra enation leaf curl virus, Barnyard virus barnyard, Citrus endogenous paretrovirus, Tiamat virus, Natevirus nate, Myrnavirus Myrna*, Betabaculovirus chofumeferanae*, Minunavirus, *Orthohantavirus oxbowaense, Bracoviriform facetosae*, Holloway virus, Berkeley virus, and Kuma virus. When compared with NR viruses, many of them had no similarities except for *P. vulgaris* alpha endornavirus 2 and Physalis Rugose Mosaic Virus. When compared against the NCBI NR viral database, the majority of sequences detected in this study showed no significant similarity to known viruses, except *P. vulgaris* alpha endornavirus 2 (98% identity) and Physalis rugose mosaic virus (78% identity). This finding suggests the presence of potentially novel or highly divergent viruses infecting common bean, reflecting the underexplored nature of bean viromes in smallholder farming systems. The limited number of matches also highlights a persistent gap in reference databases, which remain biased toward well‐studied crops and regions [28]. Previous studies have similarly reported high proportions of unclassified or unassigned viral sequences in metagenomic and metatranscriptomic datasets, underscoring the importance of HTS in revealing hidden viral diversity [29]. The detection of *P. vulgaris* alpha endornavirus 2, a commonly reported persistent virus in beans, further confirms the sequencing approach, while the identification of Physalis rugose mosaic virus expands its known host range and ecological distribution. Together, these results emphasize the urgent need for broader sampling, genome‐level characterization, and integration of newly identified viral sequences into public databases to improve virus discovery and disease management in common bean production systems.

BCMNV remains one of the major constraints to common bean production in Kenya, especially in the highlands of western Kenya, where it is highly prevalent. The virus has caused yield losses of up to 100%, posing a significant threat to food security and smallholder farmers’ livelihoods. Recent studies have provided valuable insights into its distribution, pathogenicity, and resistance in regional cultivars, which are crucial for developing effective management strategies. Surveys consistently show that BCMNV is more common and occurs at higher frequencies than BCMV [4, 9]. Its prevalence varies across different agro‐ecological zones, reaching as high as 17.9% in some areas [9]. Pathogenic characterization has identified several pathogroups, including newly discovered groups IV and VII, with BCMV and BCMNV mixed infections being widespread and complicating control efforts [30]. Screening of bean cultivars has shown variable responses, with only a limited number displaying resistance [31, 32]. The presence of resistance genes in local bean genotypes has been confirmed [30], although their effectiveness is threatened by BCMNV’s ability to overcome I‐gene resistance. This is further supported by recent work, which documented severe infection levels and high incidence rates of BCMNV in Western Kenya [33]. These outcomes align with previous reports [14] and highlight the persistence of the virus in smallholder systems. Genomic research is advancing our understanding of BCMNV evolution, revealing purifying selection, evidence of recombination, and the first full Kenyan genome sequence [34]. The high relative abundance of *Potyvirus phaseoli* (BCMNV) in Busia county suggests region‐specific conditions or host plant types that may be more susceptible to this virus, potentially due to cultivation methods or the prevalent crop types in the area. Overall, these studies demonstrate that BCMNV is firmly established in Western Kenyan bean production systems, with the potential to undermine genetic resistance and cause significant yield losses. Together, these findings emphasize the urgent need for improved seed systems, durable resistance breeding, and integrated management practices to ensure the sustainability of bean production in smallholder farms across Western Kenya.

Tomato leaf curl Cameroon alphasatellite virus detected is reported as a major pathogen affecting tomato crops in Cameroon and has been linked with several begomoviruses. Recent research has revealed that multiple begomoviruses, including Tomato leaf curl Cameroon virus and Tomato leaf curl Mali virus, are associated with the leaf curl symptoms in tomatoes. These viruses are mainly spread by the whitefly *Bemisia tabaci* and possess single‐stranded DNA genomes that are typically around 2.7–2.8 kb in size [35]. The alphasatellite associated with tomato leaf curl disease has been identified as a 1.4‐kbp fragment, highlighting its involvement in its etiology [35]. Alphasatellites endemic to Cameroon exhibit significant diversity and can infect various plant species beyond tomatoes [36]. The presence of these viruses in common beans is likely to enhance the virulence of the associated begomoviruses, complicating its management [37].

*Phaseolus vulgaris* endornavirus 1 (PvEV‐1) belongs to the *Endornaviridae* family. This virus has previously been reported in common bean fields in East Africa and Slovakia [38–40], suggesting it is widely distributed and may thrive in diverse environments. It could also potentially coinfect with other endornaviruses. The virus’s detection in common beans aligns with findings by Wainaina et al. [7], who identified it in the Western Highlands of Kenya. Known for its persistent nature, PvEV‐1 does not cause visible symptoms in its host plants. It is primarily transmitted through seeds and pollen, and its classification is reinforced by genomic studies that reveal its distinct molecular features and evolutionary connections to other picornaviruses. With a genome size of approximately 13,908 base pairs, PvEV‐1 encodes a polyprotein of 4496 amino acids [41]. Genomic comparisons show that PvEV‐1 shares significant sequence similarity with *Oryza sativa* endornavirus, indicating a close evolutionary relationship within the *Endornaviridae* family [41]. Although PvEV‐1 is generally considered a mutualistic virus that may contribute to plant resilience, its widespread presence in various bean cultivars raises important questions about its ecological role. Additional research is necessary to assess its impact on crop health, particularly across different environmental conditions [39, 42].

*Physalis Rugose Mosaic virus* (PhyRMV) is a serious pathogen that affects *Physalis peruviana*, causing significant symptoms and economic losses. The virus is primarily transmitted through direct leaf contact, pruning tools, and contaminated soil, where it can remain infectious for up to 90 days [43]. Infections can severely hinder plant growth, with studies indicating reductions of up to 70.9% in plant height and 66.4% in yield, particularly when there are coinfections with other viruses [44]. Molecular studies have shown that PhyRMV has a genome of 4162 nucleotides and shares a high level of identity with other sobemoviruses. However, it is unlikely to be transmitted via seeds [45]. The primary mechanisms of transmission include leaf contact, where direct contact between infected and healthy leaves spreads the virus, and pruning, where contaminated tools can transfer the virus between plants. Additionally, PhyRMV can persist in contaminated soil for extended periods, which further facilitates its spread. The impact of PhyRMV on plant health is significant. The association of common bean with this virus raises concerns about the possible establishment of synergistic reactions with other viruses. Infected plants may likely exhibit stunted growth and malformations, coupled with drastic reductions in fruit yield and quality. In cases of double infections, the damage can be so severe that it may result in complete crop failure.

CMV is a major pathogen that affects a wide variety of plants globally. It belongs to the genus *Cucumovirus* within the *Bromoviridae* family and is known for its extensive host range, infecting a diverse array of crops, ornamentals, and weeds. The virus possesses a tripartite RNA genome, consisting of three RNA segments, which are crucial for its replication and ability to cause disease in host plants [46]. Over the years, numerous strains of CMV have been identified, showing significant genetic variation among them [47]. In Kenya, CMV has been detected in several bean‐growing regions across East Africa, with notable reports from Kenya [48, 49], providing support for the findings of this current study. The virus causes substantial yield losses, particularly in susceptible plants, which makes its control a significant challenge for agricultural production [50].

Okra Enation Leaf Curl Virus (OELCV) detected in the transcriptome is a major begomovirus belonging to the Geminiviridae family that affects okra (*Abelmoschus esculentus*) and is transmitted by the whitefly *Bemisia tabaci*. This virus represents a serious threat to okra production, particularly in India, where it has been reported in several states. In addition, it has been detected in various regions of India, with genetic studies showing high homology among isolates from different areas, such as Salem and Madurai in Tamil Nadu [51, 52]. The coat protein gene of the virus is a region of genetic variability and plays a key role in identifying and classifying different strains of OELCV [52]. It has been associated with systemic infections in okra, leading to reduced yield and quality [51]. The disease also affects the phytochemical content of the host, negatively impacting its overall health and market value [53]. The discovery of OELCV in the common bean is likely to constrain production by causing severe symptoms such as leaf curling, stunting, and reduced flowering, which hinder photosynthesis and plant vigor, ultimately leading to decreased yield and quality [53, 54].

The relative abundance of these viruses, based on the reads, varied across different sample locations. *Potyvirus phaseoli* had the highest abundance in Busia region. The presence of *Potyvirus phaseoli* in this county may be influenced by a combination of environmental factors, such as host density, climate, and seed transmission, as well as socioeconomic factors like agricultural practices and global seed trade [55, 56]. Effective management strategies, including integrated pest control and fostering plant biodiversity, are essential for mitigating the virus’s impact on crops [57]. *Phaseolus vulgaris endornavirus 1* was most prevalent in the Vihiga/Kakamega samples. The prevalence of *P. vulgaris* endornavirus 1 (PvEV1) in the Kakamega and Vihiga counties is triggered by seed transmission, agricultural practices like replanting seeds carrying the virus, and environmental factors such as temperature, rain, and coinfection with other viruses [14, 39]. These factors and potential mutualistic roles of the virus facilitate the persistence and spread of the virus in the region [58]. Studies by Che et al. [59] highlighted the prevalence of several viruses in *Chieh-qua*, including melon yellow spot virus (MYSV), cucurbit chlorotic yellows virus (CCYV), Chieh‐qua Endornavirus (CqEV), and cucurbit chlorotic virus (CuCV), with MYSV being the most prevalent. Although this study focused on a different plant species, it underscores the potential for multiple viral infections within a single host, a factor that could complicate disease management and require further investigation into viral coinfections.

Analysis of metagenomic data revealed significant heterogeneity in viral taxa, including various species, genera, and families. A total of 93 viral species were identified, distributed across 30 classified genera and 47 unclassified genera. The analysis also uncovered 16 recognized viral families, along with 48 unclassified families, highlighting the vast and largely unexplored diversity of viral entities. Among the detected DNA viruses, the genus *Badnavirus* species were the most numerous and were observed in common beans in the region, some of which have not been previously documented to infect this crop, except for the *Pelargonium vein-banding virus*, which was reported by Wainaina et al. [7] in the Western Highlands of Kenya. These included *Badnavirus betacolocalasiae*, *Badnavirus phirubi*, *Badnavirus venatheobromae*, Jujube‐associated *Badnavirus*, Pandanus *Badnavirus*, Paper mulberry vein‐banding virus, and Stilbocarpa mosaic bacilliform virus. The importance of plant viruses in the *Badnaviridae* family is underscored by their impact on agricultural yields, as they can infect a wide range of economically important crops, leading to significant yield losses and posing threats to crop management and food security. *Badnaviruses* have been linked to declines in banana, yam, and cocoa yields [60, 61], with *Banana streak virus* (BSV) alone capable of causing over 89% production losses in banana plantations [62]. The genetic variability of *Badnaviruses* offers valuable insights into the phylogenetic relationships of crops like bananas and their evolutionary trajectories.

The variation in the relative abundance of different viruses across the Bungoma, Busia, and Vihiga/Kakamega samples underscores the complex dynamics of viral diversity in these regions. In particular, *Punavirus* was most prevalent in the Bungoma samples, while *Cadabanvirus R24* and *Orthohantavirus oxbowaense* were more abundant in the Busia and Vihiga/Kakamega samples. This variation can be attributed to several factors, including probably ecological conditions, host–virus interactions, and the methods utilized for detecting the viruses. In the Bungoma region, the local ecological conditions may be particularly favorable for the proliferation of the *Punavirus*, making it more prevalent compared to other areas. The composition of host species in Bungoma could also play a significant role in this, as certain plants or animals in the region may be more susceptible to the virus. In contrast, the higher relative abundance of *Cadabanvirus R24*, *Badnarviruses*, and *Orthohantavirus oxbowaense* in the Busia and Vihiga/Kakamega samples might be linked to environmental factors, including anthropogenic disturbances that alter the community structures of host species, as seen in other research [57]. Besides, advanced detection methods, such as multiplex RT‐PCR, have enhanced the ability to identify these viruses, revealing their prevalence in these regions [63]. These findings highlight the distinct viral profiles across different regions.

In general, the study reveals a diverse virome of⁠ both known and⁠ novel viruses, with frequent mixed infections posing serious risks to bean yields and farmer livelihoods. These findings underscore the need for integrated management strategies that combine broad‐spectrum measures, such as resistant varieties and vector control, with site‐specific interventions targeting region‐endemic viruses. The diversity observed also signals potential viral evolution, reinforcing the importance of continuous surveillance and research to protect bean production.

In addition to plant viruses, viruses of public health concern including zoonotic and human pathogens, such as the Bunyamwera virus, dengue virus, hantaviruses, and coronaviruses, as well as unclassified viruses like those in the Bunyaviridae family. Equally observed were bacteriophages comprising those infecting Pseudomonas, Streptomyces, and Mycobacteri⁠um, playing critical roles in regulating bacte⁠rial populations, shaping microbial diversity, and influencing ecosystem processes such as nutrient cycling [64, 65]. Their ecological and therapeu⁠tic potential is significant, w⁠ith a⁠pplic⁠ations in treating antibiotic‐resistan⁠t infections and promoting plant health as sustainable alternatives to pesticid⁠es [66–69]. However, li⁠mitations⁠ such as narrow host ranges, environme⁠ntal instability, regulatory hurdl⁠es, and risks of antimicrobial resistance gene dissemination remain important challenges [69, 70]. A limitation of this study is the lack of qRT‐PCR validation for the presence of these plant viruses due to resource constraints.

## 5. Conclusions

The discovery of DNA viruses previously unreported in common bean fields in Kenya, such as *Bracoviriform facetosae*, *Tomato leaf curl Cameroon alphasatellite*, *Pandoravirus dulce*, Cotton *leaf curl Burewala virus*, *Cotesia sesamiae bracovirus* and *Mimivirus* sp amongst others. The identification of these viruses raises concerns about crop health, while also opening the door for research into the potentially beneficial interactions between certain viruses and common beans, which could lead to the development of innovative agricultural practices.

The detection of RNA viruses previously unreported in common bean fields in Kenya, such as *Physalis rugose mosaic virus*, *Maize suscal virus*, *Okra enation leaf curl virus*, and *Citrus endogenous pararetrovirus.* While the detection of these viruses presents challenges for common bean production, it also opens avenues for research that could lead to improved agricultural practices and crop resilience. Understanding the dynamics of viral infections is essential for sustainable farming in the region. The results underscore the diverse and complex viral landscape impacting common beans, revealing a wide array of viruses with varying relative abundance across different regions.

## 6. Recommendation

This study provides a large repertoire of knowledge on Common beans that will inform policy decisions and common bean breeding programs in Kenya and worldwide. In addition, the outcome highlights the need for continued surveillance and research to understand the ecological roles of these viruses and their impact on crop health, Moreover, the evolving nature of viral threats to common bean production necessitates improved diagnostic and management strategies.

## Conflicts of Interest

The authors declare no conflicts of interest.

## Author Contributions

Aggrey Keya Osogo contributed to writing–original draft, visualization, project administration, methodology, investigation, formal analysis, data curation, and conceptualization. Francis Muyekho performed writing–review & editing, supervision, funding acquisition, and conceptualization. Hassan Were carried out writing–review & editing, validation, supervision, and conceptualization. Patrick Okoth contributed to writing–original draft, visualization, project administration, investigation, formal analysis, data curation, and conceptualization.

## Funding

This research received no external funding.

## Supporting Information

Additional supporting information can be found online in the Supporting Information section.

Additional supporting information is included in this article:

## Supporting information


**Supporting Information 1** 1. SM1: Table 1: Quality statistics of the sequence reads.

**Supporting Information 2** 2. SM2: Common bean viruses found in the collected samples, along with their corresponding unigenes mapped to the NCBI database, including their viral genera and families.

**Supporting Information 3** 3. GPS coordinates of the areas sampled in Western Kenya.

## Data Availability

The sequences used for this analysis can be accessed using the link https://www.ncbi.nlm.nih.gov/sra/PRJNA1226238 and the supporting information.
