# Supplementary material for: Unveiling Common Bean (Phaseolus vulgaris L) RNA‐ and DNA‐Based Virome in Western Kenya: Insights From Metatranscriptomic and Metagenomic Signatures
Source: Adv Virol. 2025 Oct 29;2025:6690945. doi: 10.1155/av/6690945 (PMC12570046; doi:10.1155/av/6690945)
Supplement: Supplementary file 1 — Supporting Information 1 1. SM1: Table 1: Quality statistics of the sequence reads. [file AV-2025-6690945-s002.docx]

**Supplementary Material 1**: **Table 1**: Quality statistics of the reads

| SAMPLE | RAW_BASE(G) | CLEAN_BASE(G) | CLEAN_Q20(%) | CLEAN_Q30(%) | CLEAN_GC(%) | EFFECTIVE(%) |
| --- | --- | --- | --- | --- | --- | --- |
| DVK1 | 17.9 | 17.72 | 97.5 | 92.98 | 41.65 | 99.02 |
| DBU1 | 11.38 | 11.21 | 97.09 | 92.01 | 42.67 | 98.53 |
| DBGM1 | 13.56 | 9.65 | 96.51 | 90.83 | 41.52 | 71.15 |
