# Supplementary material for: Unveiling Common Bean (Phaseolus vulgaris L) RNA‐ and DNA‐Based Virome in Western Kenya: Insights From Metatranscriptomic and Metagenomic Signatures
Source: Adv Virol. 2025 Oct 29;2025:6690945. doi: 10.1155/av/6690945 (PMC12570046; doi:10.1155/av/6690945)
Supplement: Supplementary file 2 — Supporting Information 2 2. SM2: Common bean viruses found in the collected samples, along with their corresponding unigenes mapped to the NCBI database, including their viral genera and families. [file AV-2025-6690945-s003.docx]

**Supplementary Materials 2:** Common bean viruses found in the collected samples, along with their corresponding unigenes mapped to the NCBI database, including their viral genera and families.

|  | **VIRUS SPECIES** | **GENERA** | **UNIGENES** | | | **FAMILY** |
| --- | --- | --- | --- | --- | --- | --- |
|  |  |  | **DVK1** | **DBU1** | **DBGM1** |  |
| 1 | Badnavirus betacolocalasiae | Badnavirus;s | 58.925778 | 0 | 0 | Caulimoviridae |
| 2 | Badnavirus phirubi | Badnavirus;s | 33.104216 | 0 | 0 | Caulimoviridae |
| 3 | Badnavirus venatheobromae | Badnavirus;s | 18.046191 | 0 | 0 | Caulimoviridae |
| 4 | Jujube associated badnavirus | Badnavirus;s | 51.560055 | 0 | 0 | Caulimoviridae |
| 5 | Pandanus badnavirus | Badnavirus;s | 85.581874 | 0 | 0 | Caulimoviridae |
| 6 | Paper mulberry vein-banding virus | Badnavirus;s | 171.40291 | 0 | 0 | Caulimoviridae |
| 7 | Pelargonium vein banding virus | Badnavirus;s | 30.365742 | 0 | 0 | Caulimoviridae |
| 8 | Stilbocarpa mosaic bacilliform virus | Badnavirus;s | 36.912123 | 70.04472 | 30.18612 | Caulimoviridae |
| 9 | Caulimovirus maculatractylodei | Caulimovirus;s | 52.58102 | 0 | 0 | Caulimoviridae |
| 10 | Lampyris noctiluca errantivirus 1 | Errantivirus;s | 17.247559 | 4.052174 | 0 | Metaviridae |
| 11 | Avian myeloblastosis virus | Alpharetrovirus;s | 188.56439 | 153.2635 | 192.1717 | Retroviridae |
| 12 | Atrato Retro-like virus | Unclassified;s | 12.908998 | 0 | 0 | Unclassified |
| 13 | Okra enation leaf curl virus | Begomovirus;s | 124.23145 | 137.4403 | 251.8785 | Geminiviridae |
| 14 | Pseudomonas phage PfAC02b | Unclassified;s | 40.274563 | 29.03169 | 16.66417 | Inoviridae |
| 15 | Mitoviridae sp. | Unclassified;s | 823.21434 | 1621.365 | 1157.1 | Mitoviridae |
| 16 | Chamaemelum virus 1 | Unclassified;s | 54.141748 | 32.7727 | 51.21974 | Rhabdoviridae |
| 17 | Acanthocystis turfacea Chlorella virus Canal-1 | Chlorovirus;s | 32.331325 | 42.75903 | 110.0569 | Phycodnaviridae |
| 18 | Dishui lake phycodnavirus 1 | Prasinovirus;s | 264.96207 | 337.7889 | 380.7092 | Phycodnaviridae |
| 19 | Dishui Lake phycodnavirus 3 | Prasinovirus;s | 47.54477 | 49.70268 | 136.7552 | Phycodnaviridae |
| 20 | Fadolivirus algeromassiliense | Fadolivirus;s | 95.794639 | 181.4572 | 102.5615 | Mimiviridae |
| 21 | Megavirus powaiense | Megavirus;s | 33.769492 | 29.81383 | 48.01786 | Mimiviridae |
| 22 | Moumouvirus goulettemassiliense | Moumouvirus;s | 32.323203 | 1.650886 | 27.29109 | Mimiviridae |
| 23 | Tupanvirus deep ocean | Tupanvirus;s | 34.522122 | 0 | 23.96737 | Mimiviridae |
| 24 | Edafosvirus sp. | Unclassified;s | 5.4919859 | 70.96636 | 39.99401 | Mimiviridae |
| 25 | Harvfovirus sp. | Unclassified;s | 32.735212 | 0 | 33.77272 | Mimiviridae |
| 26 | Hyperionvirus sp. | Unclassified;s | 55.779532 | 12.75173 | 71.07116 | Mimiviridae |
| 27 | Dasineura jujubifolia toursvirus 2a | Toursvirus;s | 267.26143 | 15.32228 | 0 | Ascoviridae |
| 28 | Diabrotica toursvirus 3a | Toursvirus;s | 128.28401 | 0 | 0 | Ascoviridae |
| 29 | Toursvirus dptv1a | Toursvirus;s | 110.60141 | 0 | 0 | Ascoviridae |
| 30 | Erythrocytic necrosis virus | Unclassified;s | 62.911096 | 44.76771 | 33.03853 | Iridoviridae |
| 31 | Golden Marseillevirus | Marseillevirus;s | 28.20209 | 129.2041 | 130.0706 | Marseilleviridae |
| 32 | Lausannevirus | Unclassified;s | 32.042132 | 28.23015 | 41.44834 | Marseilleviridae |
| 33 | Marseillevirus LCMAC202 | Unclassified;s | 74.201304 | 19.06773 | 88.26961 | Marseilleviridae |
| 34 | Yokapox virus | Centapoxvirus;s | 43.582988 | 35.44432 | 61.03503 | Poxviridae |
| 35 | Picornavirales sp. | Unclassified;s | 19.578217 | 40.37009 | 15.16739 | Unclassified |
| 36 | Bos-associated insect adintovirus | Unclassified;s | 97.4254 | 66.86087 | 32.98441 | Adintoviridae |
| 37 | Bos-associated insect adintovirus 2 | Unclassified;s | 90.098197 | 72.21937 | 34.71799 | Adintoviridae |
| 38 | Ladona dragonfly adintovirus | Unclassified;s | 202.5109 | 169.8214 | 85.60608 | Adintoviridae |
| 39 | Megastigmus wasp adintovirus | Unclassified;s | 198.33886 | 109.8709 | 65.10537 | Adintoviridae |
| 40 | Monosiga MELD virus 2 | Unclassified;s | 35.107648 | 34.36774 | 41.42682 | Adintoviridae |
| 41 | Strongylocentrotus sea urchin adintovirus | Unclassified;s | 7.3226478 | 53.9579 | 0 | Adintoviridae |
| 42 | Gammatectivirus GC1 | Gammatectivirus;s | 66.625781 | 0 | 0 | Tectiviridae |
| 43 | Drosophila suzukii associated hytrosavirus 1 | Unclassified;s | 26.72039 | 19.48264 | 43.61378 | Hytrosaviridae |
| 44 | Hemigrapsus takanoi nimavirus | Unclassified;s | 15.458923 | 4.889162 | 4.329836 | Nimaviridae |
| 45 | Cotesia sesamiae bracovirus | Bracoviriform;s | 37.782831 | 38.81469 | 55.64194 | Polydnaviriformidae |
| 46 | Cotesia vestalis bracovirus | Bracoviriform;s | 15.086419 | 0 | 0 | Polydnaviriformidae |
| 47 | Apophua simplicipes ichnovirus | Ichnoviriform;s | 86.059985 | 24.01018 | 15.66776 | Polydnaviriformidae |
| 48 | Pandoravirus massiliensis | Pandoravirus;s | 7.1716654 | 45.49296 | 60.05973 | Unclassified |
| 49 | Pandoravirus quercus | Pandoravirus;s | 15.518381 | 12.25783 | 16.56162 | Unclassified |
| 50 | Aedes aegypti To virus 1 | Unclassified;s | 12.534262 | 24.29479 | 0 | Unclassified |
| 51 | Aphanizomenon phage Yong-DA | Unclassified;s | 15.763532 | 21.73802 | 0 | Unclassified |
| 52 | Bacteriophage sp. | Unclassified;s | 23.10001 | 30.08949 | 20.8225 | Unclassified |
| 53 | Ips errant-like virus 3 | Unclassified;s | 4.9689396 | 21.88898 | 31.66192 | Unclassified |
| 54 | Prokaryotic dsDNA virus sp. | Unclassified;s | 42.636405 | 7.485696 | 3.383717 | Unclassified |
| 55 | uncultured marine phage | Unclassified;s | 27.114591 | 176.3209 | 41.84283 | Unclassified |
| 56 | uncultured marine virus | Unclassified;s | 8.4321399 | 22.28696 | 51.17079 | Unclassified |
| 57 | Crassvirales sp. | Unclassified;s | 5.129228 | 142.349 | 38.51958 | Unclassified |
| 58 | Hagravirus HGTV1 | Hagravirus;s | 4.2547495 | 152.192 | 35.63177 | Halomagnusviridae |
| 59 | Bucovirus buco | Bucovirus;s | 2.2808247 | 36.17064 | 0 | Autographiviridae |
| 60 | Drulisvirus altogao | Drulisvirus;s | 11.311407 | 13.952 | 4.118624 | Autographiviridae |
| 61 | Klebsiella phage vB_Kpn_K15PH90 | Drulisvirus;s | 28.39394 | 18.34505 | 12.06169 | Autographiviridae |
| 62 | Koutsourovirus KDA1 | Koutsourovirus;s | 22.388785 | 28.17891 | 0 | Autographiviridae |
| 63 | Pseudomonas phage PSV6 | Pifdecavirus;s | 9.6426946 | 38.83668 | 5.01575 | Autographiviridae |
| 64 | Escherichia phage vB_EcoP_PAS7 | Unclassified;s | 334.12783 | 425.3327 | 58.26308 | Autographiviridae |
| 65 | Herelleviridae sp. | Unclassified;s | 0 | 574.3119 | 136.3361 | Herelleviridae |
| 66 | Ochrobactrum phage vB_OspM_OC | Unclassified;s | 21.014474 | 0 | 3.957741 | Kyanoviridae |
| 67 | Synechococcus phage S-BM3 | Unclassified;s | 14.428328 | 121.6703 | 3.752524 | Kyanoviridae |
| 68 | Jiaodavirus kppv15 | Jiaodavirus;s | 26.373448 | 29.33751 | 7.641871 | Strabovirida |
| 69 | Klebsiella phage KP17 | Jiaodavirus;s | 12.276204 | 37.35719 | 0 | Strabovirida |
| 70 | Klebsiella phage ValerieMcCarty01 | Jiaodavirus;s | 12.985495 | 6.537507 | 0 | Strabovirida |
| 71 | Flavobacterium phage FPSV-S29 | Fipvunavirus;s | 0 | 134.9913 | 14.42822 | Unclassified |
| 72 | Lanavirus lana | Lanavirus;s | 21.967943 | 67.74062 | 9.998502 | Unclassified |
| 73 | Xanthomonas phage XbC2 | Mimasvirus;s | 9.0934842 | 36.8536 | 66.22102 | Unclassified |
| 74 | Streptomyces phage GirlPower | Rimavirus;s | 17.169272 | 0 | 0 | Unclassified |
| 75 | Rosemountvirus SE13 | Rosemountvirus;s | 31.999971 | 19.61252 | 0 | Unclassified |
| 76 | Streptomyces phage RosaAsantewaa | Scapunavirus;s | 25.948907 | 0 | 0 | Unclassified |
| 77 | epukevirus Psa21 | Tepukevirus;s | 13.146171 | 38.60733 | 13.96116 | Unclassified |
| 78 | Arthrobacter phage Sporto | Unclassified;s | 13.768103 | 0 | 0 | Unclassified |
| 79 | Bacillus phage vB_BceM-HSE3 | Unclassified;s | 65.7809 | 17.54416 | 43.38118 | Unclassified |
| 80 | Brevundimonas phage vB_BgoS-Bajun | Unclassified;s | 13.250506 | 70.04472 | 12.06169 | Unclassified |
| 81 | Caudoviricetes sp. | Unclassified;s | 346.85507 | 694.6703 | 259.9851 | Unclassified |
| 82 | Caulobacter phage Cr30 | Unclassified;s | 30.029887 | 8.551972 | 15.55751 | Unclassified |
| 83 | Idiomarinaceae phage Phi1M2-2 | Unclassified;s | 47.683157 | 8.056787 | 26.16197 | Unclassified |
| 84 | Microbacterium phage Big4 | Unclassified;s | 0 | 0 | 16.22089 | Unclassified |
| 85 | Microbacterium phage Count | Unclassified;s | 12.958215 | 0 | 211.9266 | Unclassified |
| 86 | Phage AS32 | Unclassified;s | 6.4337715 | 0 | 0 | Unclassified |
| 87 | Siphoviridae sp. ctnLs3 | Unclassified;s | 40.1517 | 36.19761 | 33.99938 | Unclassified |
| 88 | Stenotrophomonas phage vB_SmaS-DLP_6 | Unclassified;s | 17.506463 | 12.98843 | 0 | Unclassified |
| 89 | uncultured Caudovirales phage | Unclassified;s | 779.55268 | 3259.73 | 727.2775 | Unclassified |
| 90 | uncultured Mediterranean phage uvDeep-CGR2-KM21-C88 | Unclassified;s | 0 | 68.73548 | 47.34493 | Unclassified |
| 91 | uncultured Mediterranean phage uvMED | Unclassified;s | 10.360768 | 86.06559 | 4.041948 | Unclassified |
| 92 | Virus Rctr197k | Unclassified;s | 1.9595818 | 10.35873 | 33.89164 | Unclassified |
| 93 | Xanthomonas phage NEB7 | Unclassified;s | 0 | 0 | 414.1344 | Unclassified |
